# Food Insecurity and Type 2 Diabetes Among Latinos: Examining Neighborhood Cohesion as a Protective Factor

**DOI:** 10.1007/s40615-022-01386-4

**Published:** 2022-08-11

**Authors:** Brandon Osborn, Brittany N. Morey, John Billimek, Annie Ro

**Affiliations:** 1 Department of Health, Society, and Behavior, Program in Public Health, University of California, Irvine, Irvine, USA; 2 Department of Family Medicine, School of Medicine, University of California, Irvine, Irvine, USA

**Keywords:** Food insecurity, Latinos, Diabetes, Psychosocial, Social cohesion, Neighborhoods, Social support, 62P25

## Abstract

Qualitative work has found that Latino food pantry recipients share food and reciprocally provide social support to their food-insecure neighbors. These findings suggest that neighborhood cohesion (NC) may serve as an important community-level resource that Latinos utilize as a coping mechanism when food-insecure. High levels of NC may be a proxy for instrumental support outside the household and act as a buffer against the adverse health effects of food insecurity including type 2 diabetes (T2D), which is highly sensitive to food insecurity. The purpose of this study was to quantitatively test this theory by examining whether NC moderated the association between T2D and food security (FS) status among Latino adults nationwide. We used data from the 2013–2018 National Health Interview Survey (*n* = 23,478). We found that FS status was associated with T2D prevalence, with Latino adults having a higher odds of T2D if they had low FS or very low FS compared to their FS counterparts. We also found Latinos adults who reported high NC had a lower odds of T2D compared to those who reported low NC. However, we did not find there was significant interaction between FS status and NC on T2D. NC may instead be a precursor to FS status, rather than a buffer of food insecurity on T2D. Low NC may lead to less instrumental support and tangible benefits that determine FS. Additionally, perceived NC might not align with objective NC and T2D may be too distal of a health outcome to test the protective effect of NC.

## Introduction

### Food Insecurity and Type 2 Diabetes

There are multiple factors that increase risk for type 2 diabetes (T2D) and T2D-related disparities in the USA including ancestry and genetic, behavioral, environmental, and social determinants [1]. Of these, food insecurity, or the lack of access to enough food for an active, healthy life, is a particularly important risk factor for T2D [2–5]. The relationship between food insecurity and T2D has been consistently established in cross-sectional studies across different samples and geographical regions [4–8]. This is especially evident by the higher prevalence of diabetes of people living in households that are food-insecure compared to those living in food-secure households. One study showed a 3% difference in diabetes prevalence among low-income adults living in food-insecure households (10.2%) compared to those in food-secure households (7.4%) in the USA [9]. In addition, in the USA and Canada, food-insecure adults of different races/ethnicities are two to three times more likely to have T2D compared to their food-secure counterparts, even after adjusting for other risk factors such as lifestyle factors, income, employment, and physical measures [7, 8]. The link between food insecurity and T2D is often attributed to irregular eating patterns [4, 8, 10] which is associated with increased weight and insulin resistance [11]. Individuals with prolonged food insecurity also experience chronic stress [12], which may result increased adiposity, a precursor to T2D [13]. Food insecurity experienced as a chronic stressor promotes a stress response, which may result in compensatory eating behaviors, such as the selection of energy-dense foods over fresh produce, which increases glycemic loads and risk of T2D [14, 15].

There are distinct health disparities among different racial/ethnic populations in the USA when examining food insecurity and its relation to T2D. While there are multiple factors that result in diabetes-related disparities among Latinos including ancestry and genetic risk, behavioral, environmental, and social determinants [1], Latino adults are disproportionately affected by T2D, with a higher prevalence of diagnosed T2D (12.5%) compared to non-Hispanic whites (7.5%) in 2017–2018 [16]. Additionally, the prevalence of food insecurity is considerably higher among Latino households (17.2%) than the national average (10.5%) in 2020 [17]. The association between food insecurity and T2D is also stronger among Latinos compared to other racial/ethnic groups, with Latinos being more likely to have T2D when food insecure compared to other racial/ethnic groups, underscoring the importance of examining this association in depth [2, 7, 8]. Latina adults experiencing very low food security have been found to be 3.3 times more likely to have T2D compared to their food secure or low food secure counterparts after controlling for age, employment status, acculturation, waist circumference, and lifestyle characteristics [8]. Other racial/ethnic groups do not exhibit such a robust association between food insecurity and T2D [18].

### Neighborhood Social Cohesion as a Moderator

Examining factors that can disrupt the association between food insecurity and T2D can better inform how to address the adverse effects of food insecurity, especially among Latinos. For instance, several studies have established that psychosocial factors can buffer the negative influence of food insecurity on health outcomes. One study found a buffering effect of *social support* against food insecurity’s association with depression among Latinos adults with T2D [19]. Among Latino adults, the predicted probability of having depression with low social support and having high food insecurity was above 0.8, whereas the predicted probability of having depression with high school support and having high food insecurity was less than 0.1 [19]. This buffering effect may arise from family, friends, and others providing resources to those experiencing food insecurity protect mental health such as food, money, and/or emotional support [20], which can lessen negative emotions associated with food insecurity that lead to behavior responses, such as compensatory eating behaviors [21].

Community-level psychosocial resources, such as neighborhood social cohesion, can likewise be important factors at the intersection of T2D [22] and food security [23–26]. *Social cohesion* is a concept that suggests that trusting relationships in a community yield important resources that can be tapped by community members and used for goods such as food and services such as transportation. Social cohesion is often conceptualized as occurring within a set geographic place, most often in one’s neighborhood. Neighborhood social cohesion, or neighborhood cohesion, can be measured at the individual level (individual perception of neighborhood social cohesion), the neighborhood (average magnitude of individual-level perceptions of neighborhood social cohesion), or both [27–29]. Neighborhood cohesion, as measured by individual perception of neighborhood cohesion, has a direct association with both food insecurity and measures related to T2D. High neighborhood cohesion been found to improve safety and serve as a protective factor [28, 30] against food insecurity among low-income families living in low-income neighborhoods [27]. Other work has found higher neighborhood cohesion to be associated with lower glycohemoglobin (HbA1c) levels [31]. This suggests that neighborhood cohesion may be a protective factor against both food insecurity and T2D independently.

Trusting neighborhood relationships can help protect families from the experience of food insecurity [27] as well encourage positive health behaviors and increase access to services and amenities when faced with food insecurity [27, 30, 32]. There are at least two distinct mechanisms by which neighborhood social cohesion might buffer against the impact of food insecurity on T2D. The first mechanism is the direct sharing of services and resources among a socially cohesive group. For example, neighbors may provide direct assistance by sharing a meal, extra food, cash, or a gift card to a restaurant or grocery store. Neighbors may provide indirect assistance by referring families or individuals who are experiencing food insecurity to a local community service, such as a food pantry or food bank, to families or individuals who are experiencing food insecurity. The second mechanism is through the positive psychosocial benefits of having high neighborhood cohesion, which may help bolster resilience [27, 33]. For example, cohesion within a neighborhood can impact psychosocial processes by providing affective support and act as a source of self-esteem and mutual respect. These examples highlight the potential benefits of neighborhood cohesion within the context of food insecurity.

Some qualitative work has found neighborhood social cohesion to be an important factor in Latinos’ experiences with food insecurity. A 2017 study among food pantry recipients, who were primarily Spanish-speaking Latinos, found that recipients shared food and reciprocally provided social support to their food-insecure neighbors [34]. These findings suggest that neighborhood cohesion and social capital derived from neighborhood cohesion may serve as an important community-level resource that Latinos utilize as a coping mechanism when food insecure. This has not been tested empirically in quantitative data, however. It is also possible that neighborhood social cohesion may not be a moderator of the relationship between food insecurity and T2D, but instead a potential determinant of food security.

Understanding if high neighborhood cohesion buffers the impact of food insecurity could inform future public health policy and interventions by highlighting the importance of neighborhood interpersonal processes. In this paper, we use the National Health Interview Survey, a nationally representative sample, to determine whether neighborhood cohesion moderates the association between food security status and T2D among Latinos nationwide. We hypothesize (1) that food security status will be associated with T2D among Latino adults and (2) that neighborhood cohesion will moderate the relationship between food security status and T2D, such that high neighborhood cohesion will result in a weaker association between food security status and T2D.

## Methods

We analyzed data from the 2013–2018 National Health Interview Survey (NHIS) [35]. The NHIS is an annual, cross-sectional household interview survey conducted by the Centers for Disease Control and Prevention that gathers health-related data in a nationally representative sample of the civilian, non-institutionalized US population. The sample is selected using a complex, stratified, multistage probability cluster design. We limited our sample to respondents who self-reported Latino ethnicity, were over the age of 18, and who responded to variables of interest including covariates (*n* = 23,478). We used listwise deletion to handle missing data on our variables. The percent missing (7%) is below the range that is considered problematic (10% or more) for missing data biases [36]. All statistical analyses were conducing using Stata 14 [37].

### Measures

*Type 2 diabetes* was measured by two questions, “Has a doctor ever told you that you have diabetes?” and if respondents answered yes, the respondent is asked to specify “Type 1 or Type 2 Diabetes?.” T2D was distinguished if respondents answered “yes” to the first question and specified T2D in their response to the second question. Responses were dichotomized to either 0 (no) or yes (1).

*Food security status* was measured by utilizing the validated USDA’s 10-item adult food security module on the NHIS. Three categories were assigned according to the USDA guidelines [38]: high/marginal food security (0), low food security (1), very low food security (2). The level of food security is determined by the number of affirmative responses to the 10-item questionnaire module. An example of one of the questions is “[In the last 12 months], were you ever hungry but didn’t eat because there wasn’t enough money for food?” Respondents who provided affirmative responses to any of the items were considered fully food secure. Those who provided 0–2 affirmative responses for a household were considered to have high/marginal food security. Those who provided 3–5 affirmative responses for a household were considered to have low food security status. Those who provided 6–10 affirmative responses for a household were considered to have very low food security.

*Neighborhood cohesion* can be measured by individual perceptions of neighborhood social cohesion [27]. Scholars often use individual perceptions of neighborhood social cohesion [28, 39] as it has been found to be valid and reliable. Additionally, when trying to obtain more objective measures of neighborhood social cohesion, there is difficulty in obtaining consensus from residents about a definition and boundary for their neighborhood [27]. In this study, neighborhood cohesion was measured using four questions modified from an original scale developed by the Project on Human Development in Chicago Neighborhoods Community Survey [28]. The four items have been used in other studies to examine neighborhood cohesion and health outcomes [27, 39–42]. Participants rated agreement or disagreement on a 4-point scale (1, definitely agree; 2, somewhat agree; 3, somewhat disagree; and 4, definitely disagree) with the following 4 statements: (1) People in this neighborhood help each other out; (2) There are people I can count on in this neighborhood; (3) People in this neighborhood can be trusted; and (4) This is a close-knit neighborhood. Participant responses were reverse coded; a higher score equated to higher neighborhood social cohesion [28]. The Cronbach’s alpha of the four items was 0.89 in our sample, which indicated high reliability across the four items. A neighborhood social cohesion score was constructed by summing the responses to the questions, with a possible range of scores from 4 to 16 [41, 42]. Similar to Yi et al., we dichotomized neighborhood social cohesion as “high” if the score was at or above the median score (13 and higher) or “low” if the score below the median score [41].

*Covariates* included age (years); sex (male, female); education level (less than high school, high school diploma or equivalent, some college or technical training, university graduate or greater); poverty level (less than 1.0 of the federal poverty line, 1.00–1.99, and 2.0 and greater); having health insurance (yes, no); and nativity (US-born or foreign-born). These variables may be associated with both food security status and T2D simultaneously [1, 4, 43]. We also controlled for length of time living in the neighborhood (less than 1 year, 1–3 years, 4–10 years, 11–20 years, 20 + years) since this variable may influence perceptions of neighborhood cohesion [27, 28, 41]. Lastly, we controlled for family type (one adult and no children, multiple adults and no children, one adult and 1 + children, multiple adults and 1 + children) since the USDA adult food insecurity module does account for children and household food insecurity is more prevalent among households with children and one adult [44].

### Data Analyses

We conducted a series of logistic regressions to determine the potential interactive relationship between food security status and neighborhood cohesion to T2D. We first examined the unadjusted association between food security status and T2D (model 1) to establish the primary association between food security status and T2D. We then examined the association between food security status and T2D after controlling for covariates (model 2). We then added neighborhood social cohesion in model 3. We then included an interaction term between food security status and neighborhood social cohesion on T2D (model 4) to test whether neighborhood social cohesion moderated food security status’ association with T2D. Additionally, we conducted post hoc analyses such as adjusted Wald tests to test the statistical significance of the interaction and an *F*-test of overall significance for the interaction term. Lastly, we calculated and graphed the predicted probabilities of T2D prevalence on food security status, comparing those with high versus low neighborhood social cohesion, from the logistic regression shown in model 4.

## Results

### Sample Characteristics

The sample was 53.5% female and 44.8% native-born (Table 1). The average age of respondents was 43.0 years. The prevalence of self-reported T2D was 10.8% and a combined 15.7% of respondents had low or very low food security. A majority of respondents lived in their neighborhoods for less than 10 years (70.0%) and were below 200% of the federal poverty line (51.7%). In addition, 38.1% of respondents perceived themselves as living in a neighborhood with high cohesion.

**Table 1:** Sample description, National Health Interview Survey 2013–2018: Latino adults (*n* = 23,478)

Demographics	
Mean age	43.0
Gender, %	
Female	53.5
Male	46.5
Education, %	
Less than high school	30.8
High school diploma or equivalent	25.4
Some college or technical training	27.2
University grad +	16.5
Poverty level ratio, %	
Less than 1.0	23.6
1.0–1.99	28.1
2.0 +	48.2
Born in the USA, %	44.8
Family type, %	
One adult, no children	23.2
Multiple adults, no children	29.9
One adult, 1 + children	7.5
Multiple adults, 1 + children	39.4
Currently has health insurance, %	75.1
Food security status, %	
High/marginal food security	84.3
Low food security	9.9
Very low food security	5.8
Type 2 diabetes prevalence, %	10.8
Neighborhood characteristics	
Mean level of neighborhood cohesion (range of values: 4–16)	11.5
Length of time living in present neighborhood, %	
Less than 1 year	14.3
1–3 years	26.3
4–10 years	29.4
11–20 years	17.1
20 + years	12.8

### Multivariable Regression Results

There were significant differences in odds of T2D for those with low food security (OR = 1.64, 95% CI: 1.42–1.89) and very low food security (OR = 1.93, 95% CI: 1.62, 2.30) compared to the reference group of high/marginal food security in our unadjusted model (model 1, Table 2). People with low or very low food security had a higher odds of having T2D compared to their food-secure counterparts. The odds of T2D for respondents with low food security (AOR = 1.84, 95% CI: 1.56–2.17) and very low food security (AOR = 2.00, 95% CI: 1.64–2.43) remained significant relative to the reference group after adjusting for covariates (model 2, Table 2). Similar to model 1, people with low or very low food security had a higher odds of having T2D compared to their food secure counterparts after taking differences in age, sex, education level, poverty, having health insurance, nativity, and length of time living in the neighborhood into account. In model 3, people who reported having high neighborhood cohesion had a lower odds (AOR = 0.86, 95% CI: 0.76–0.97) of T2D compared to those who reported having low neighborhood cohesion. When neighborhood cohesion was added to the model, the odds ratios for the different food security status groups and T2D remained in the same direction as in model 2.

**Table 2:** National Health Interview Survey 2013–2018: Latino adults (*n* = 23,478). Odd ratios of having type 2 diabetes via logistic regression

	Model 1			Model 2			Model 3			Model 4		
	Unadjusted main effects of food security status			Food security status, controls			Food security status, main effects of neighborhood cohesion, controls			Interaction between food security status and neighborhood cohesion		
	OR	[95% CI]		OR	[95% CI]		OR	[95% CI]		OR	[95% CI]	
Food security status												
High/marginal food security	ref			ref			ref			ref		
Low food security	1.64**	1.42	1.89	1.84**	1.56	2.17	1.83**	1.55	2.15	1.67**	1.35	2.05
Very low food security	1.93**	1.62	2.30	2.00**	1.64	2.43	1.97**	1.62	2.4	2.01**	1.61	2.51
Neighborhood cohesion												
Low							ref			ref		
High							0.86*	0.76	0.97	0.84*	0.74	0.96
Interaction: food security status × neighborhood cohesion												
High/marginal food security, low cohesion										ref		
Low food security, low cohesion										ref		
Very low food security, low cohesion										ref		
High/marginal food security, high cohesion										ref		
Low food security, high cohesion										1.31	0.92	1.88
Very low food security, high cohesion										0.92	0.61	1.38
Covariates												
Age				1.06**	1.06	1.07	1.06**	1.06	1.07	1.06**	1.06	1.07
Gender												
Male				ref			ref			ref		
Female				0.84**	0.74	0.94	0.83**	0.74	0.94	0.84**	0.74	0.94
Family type												
One adult, no children				ref			ref			ref		
Multiple adults, no children				1.24**	1.08	1.42	1.24**	1.08	1.43	1.24**	1.08	1.43
One adult, 1 + children				0.99	0.76	1.28	0.99	0.76	1.28	0.99	0.76	1.28
Multiple adults, 1 + children				1.13	0.97	1.32	1.14	0.97	1.33	1.14	0.97	1.33
Ratio of family income to poverty threshold												
Less than 1.0				ref			ref			ref		
1.0–1.99				0.83**	0.71	0.95	0.83**	0.71	0.95	0.83**	0.71	0.95
2.0 +				0.69**	0.58	0.81	0.69**	0.59	0.81	0.69**	0.59	0.81
Year												
2013				ref			ref			ref		
2014				0.87 +	0.74	1.01	0.87 +	0.74	1.01	0.87 +	0.74	1.01
2015				0.99	0.84	1.15	0.98	0.84	1.15	0.98	0.84	1.15
2016				0.99	0.82	1.20	0.99	0.82	1.20	0.99	0.82	1.20
2017				1.00	0.83	1.20	1.00	0.83	1.20	1.00	0.83	1.20
2018				1.01	0.84	1.21	1.01	0.84	1.22	1.01	0.84	1.22
Educational attainment												
Less than high school				ref			ref			ref		
High school diploma or equiv				0.83*	0.72	0.97	0.84*	0.72	0.97	0.84*	0.72	0.97
Some college or tech				0.80**	0.69	0.93	0.81**	0.69	0.93	0.81**	0.70	0.93
Bachelor’s +				0.54**	0.44	0.65	0.54**	0.44	0.66	0.54**	0.44	0.66
Duration of living in present neighbourhood												
Less than 1 year				ref			ref			ref		
1–3 years				1.11	0.90	1.37	1.11	0.90	1.37	1.11	0.90	1.37
4–10 years				1.23*	1.01	1.51	1.24*	1.01	1.53	1.24*	1.01	1.52
11–20 years				1.34**	1.09	1.65	1.36**	1.10	1.68	1.36**	1.10	1.68
20 years +				1.19	0.96	1.48	1.21 +	0.98	1.5	1.21 +	0.97	1.50
Nativity/Born in the USA				1.50**	1.33	1.70	1.51**	1.34	1.71	1.51**	1.34	1.71
Uninsured				0.70**	0.60	0.82	0.70**	0.59	0.82	0.70**	0.59	0.82

Tests of significance: + *p* < 0.1, *p < 0.05, **p < 0.01

Model 4 included interaction terms between food security status and neighborhood cohesion. There was not a significant interaction between food security status and neighborhood social cohesion on T2D (overall *F*-test = 1.27, *p*-value = 0.28). Among respondents who reported low food security, there was no significant difference in odds of having T2D for those who reported high versus low neighborhood cohesion (AOR = 1.31, 95% CI: 0.92–1.88). Among respondents who reported very low food security, there was no significant difference in odds of T2D for those who reported high versus low neighborhood cohesion (AOR = 0.92, 95% CI: 0.61–1.38).

**Fig. 1:**
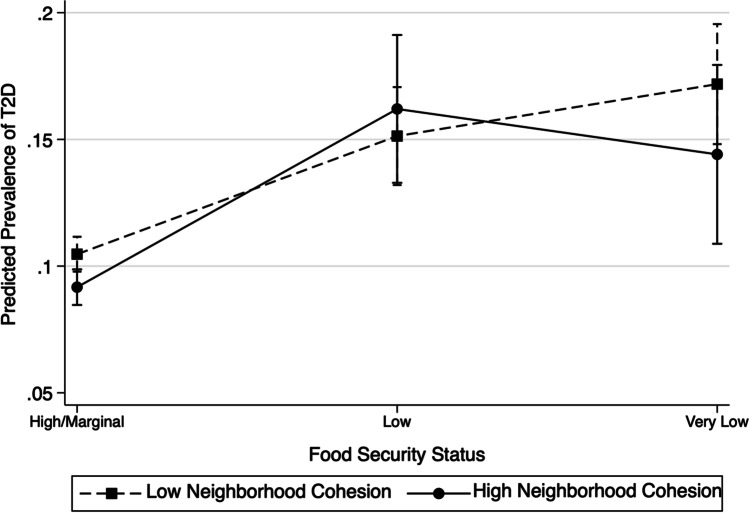
Predicted prevalence of type 2 diabetes, interaction between food security status and level of neighborhood cohesion, NHIS 2013–2018, Latinos adults (*n* = 23,478)

According to Fig. 1, those with high/marginal food security had the lowest predicted T2D probability, and this was similar at high (9.2%) and low (10.5%) neighborhood social cohesion. Those with low food security had comparably higher predicted T2D probability of 16.2% and 15.1% for those with high and low neighborhood social cohesion, respectively. Lastly, those with very low food security had a predicted T2D probability of 14.2% for high and 17.2% for low neighborhood cohesion. Those with very low food security have the highest predicted T2D probability at low neighborhood cohesion (17.2%) compared to all other groups shown, but the estimate fell within the margin of error for those with very low food security at high neighborhood cohesion. Overall, Fig. 1 illustrates that there was no statistically significant interaction between food insecurity and neighborhood cohesion such that those with high neighborhood cohesion had a weaker association between food security and T2D prevalence as those with low neighborhood cohesion.

### Sensitivity Checks

We ran our analyses on all adults (18 and older). However, we reran models 1–4 among working-age adults only (18–65 years of age) since diabetes prevalence is higher among older adults. Overall, our results did not change. We also examined a different outcome variable of self-reported T2D combined with impaired glucose tolerance to include those with self-reported prediabetes but the overall results did not change.

## Discussion

This study is the first to test whether neighborhood cohesion moderates the association between food security status and T2D among Latinos nationwide. After controlling for covariates, we found that Latinos with low food security had 1.84 times the odds of having T2D and that those with very low food security had 2.0 times the odds of having T2D compared to those who were food secure. Originally, we posited that lower food security status would be associated with more T2D prevalence, and that this association would differ by level of neighborhood cohesion such that those with higher neighborhood cohesion would be less likely to have T2D when food insecure. To our knowledge, this effect modification hypothesis has not been empirically tested with quantitative data. Although food security status was associated with T2D, neighborhood cohesion did not moderate the association. While qualitative work has found Latino individuals who are food insecure to utilize their social connections to share food and resources [34], we did not find that neighborhood social cohesion made any quantitative difference in the association between food insecurity and T2D.

Our null findings add to other work in the food insecurity literature that has examined moderators of food insecurity’s association with health outcomes among Latinos. Although we did not detect a statistically significant interaction between food insecurity and neighborhood cohesion on T2D, other studies have identified psychosocial buffers against food insecurity on metabolic health outcomes. One study, which focused on Latinos diagnosed with T2D, found that social support buffered the effect against food insecurity’s association with negative emotions [45]. Additionally, another study found maternal stress to moderate the association between food insecurity and obesity among youth [46].

One possible explanation for our null findings is that neighborhood cohesion may instead be a precursor to food insecurity, rather than a moderator/buffer of food insecurity on T2D. We found a main effect of neighborhood cohesion, such that Latinos reporting high levels of neighborhood cohesion were 0.86 less likely to have type T2D compared to their counterparts who reported low levels of neighborhood cohesion. Low neighborhood cohesion may lead to less instrumental support and tangible benefits that determine household levels of food security. Martin et al. found that community-level social capital, including neighborhood cohesion, is associated with decreased risk of hunger [26]. Another study found that higher neighborhood social cohesion is associated with higher food security among households with and without children in the USA [23]. However, there is conflicting evidence against this idea, as a different study found that neighborhood social cohesion was not associated with food security (reduction in food insecurity) [47]. Overall, our study suggests that neighborhood cohesion is an important factor that potentially affects both food insecurity and metabolic outcomes such as T2D, and its exact role warrants future study. While neighborhood cohesion may affect the actual exposure to household food insecurity, we found that it did not alter the association between food insecurity and T2D. This could be because T2D is too distal of an outcome due to the long latency period of T2D [6, 48] or because of the limitations of the self-reported measures.

This study had a number of limitations. The NHIS is cross-sectional and cannot be used to determine the causality or directionality of the models. Additionally, all measures were self-reported and thus are at risk of recall and social desirability biases [49]. T2D was measured by a respondent reporting whether a physician told them they have diabetes. This form of measurement excludes respondents who may not have access to a physician and may thus be undiagnosed. We adjusted for health insurance status to address this possibility, however. Individuals with undiagnosed T2D are less likely to have regular access to care and more likely to be low-income and represent a high proportion of the Latino population in the USA [50]. This may have resulted in underestimating Latinos with T2D, thus generating more conservative results when testing the interaction and our non-significant results.

We used perceived social neighborhood cohesion, which might not align with the actual neighborhood environment. An individual’s perception of neighborhood social cohesion and the neighborhood average magnitude of perceived social cohesion can be quite different [27]. When drawing inferences from using self-reported measures of neighborhood cohesion, researchers should keep in mind that definitions of social cohesion may be shift and not be defined spatially, but rather by social groupings such as family, affinity, ideology, and identity.

We acknowledge that we did not account for the actual neighborhood ethnic make-up in our analysis (i.e., percent foreign-born, percent Latino), but the role of this factor is unclear. Some work has found that Latinos living neighborhoods with a high immigrant composition have diets lower in fat and processed foods as well as overall better access to healthy [51] while others have found that Latino residence in immigrant enclaves report worse social environments including social cohesion [51]. Future research could use multi-level modeling to consider and control for the demographic and cultural makeup of neighborhoods, for example, by measuring the percentage of coethnicity or percentage of the immigrant population in a neighborhood. Additionally, future research should examine more potentially proximal measures of health including depression and obesity, rather than T2D.

Future interventions focusing specifically on T2D among Latinos should still consider that both neighborhood cohesion and food insecurity are important social determinants that should be targeted. Among Latinos, food insecurity is a significant risk factor for not only T2D, but also proper management of T2D [6]. Future interventions should include screening for food insecurity by healthcare providers and community- and policy-based interventions aimed at increasing food security among Latinos [6]. Neighborhood cohesion has had a significant positive independent association with glycemic control among adults with T2D [31]. Improving neighborhood cohesion may help those with T2D better control their diabetes and help prevent the onset of chronic disease among Latinos. Future public health interventions should focus on not only the built environment such as high-quality schools and access to parks, but also economic policy to bolster neighborhood characteristics that promote cohesion within a neighborhood such as home ownership.

In summary, the study results indicate that food insecurity and neighborhood cohesion are significantly associated with T2D, but neighborhood cohesion does not moderate the association between food insecurity and T2D. Perceived neighborhood cohesion might not align with the actual neighborhood environment and T2D may be too distal of a health outcome to test the protective effect of neighborhood social cohesion. These findings warrant future inquiry, including longitudinal studies, that examine the relationships between neighborhood cohesion, food insecurity, and T2D.

## Data Availability

Data is publicly available here: https://nhis.ipums.org and here: https://www.cdc.gov/nchs/nhis/data-questionnaires-documentation.htm. Lynn A. Blewett, Julia A. Rivera Drew, Miriam L. King, and Kari C.W. Williams. IPUMS Health Surveys: National Health Interview Survey, Version 6.4 [dataset]. Published online 2019.

## Code Availability

Code for statistical analyses in this study will be available upon request.
